# The Impact of Telehealth Use Experience on Recruitment of Underserved Populations Into a Telehealth-Delivered Mindfulness-Based Trial for Patients With Chronic Low Back Pain: Cross-Sectional Survey

**DOI:** 10.2196/82093

**Published:** 2026-07-29

**Authors:** Marva V Foster, Phuong Tra Nguyen, Janice Weinberg, Keturah R Faurot, Natalia Morone

**Affiliations:** 1 Department of Medicine, Section of General Internal Medicine, Boston University Chobanian and Avedisian School of Medicine, 801 Massachusetts Ave., Boston, MA, 02118, United States, 1 857-364-6056; 2 Center for Health Optimization and Implementation Research (CHOIR), VA Bedford and VA Boston Healthcare Systems, Boston, MA, United States; 3 Boston Medical Center, Boston, MA, United States; 4 Department of Biostatistics, Boston University School of Public Health, Boston, MA, United States; 5 Program on Integrative Medicine, Department of Physical Medicine and Rehabilitation, University of North Carolina at Chapel Hill, Chapel Hill, NC, United States

**Keywords:** telehealth, mindfulness-based stress reduction, low back pain, safety net, pragmatic trial, digital literacy, social determinants of health

## Abstract

**Background:**

Mindfulness-based stress reduction (MBSR) is recommended as a noninvasive, evidence-based treatment for chronic low back pain (CLBP); however, access and uptake are often constrained among medically underserved populations. Telehealth-delivered MBSR may scale access, yet little is known about the characteristics of underserved patients who have experience using telehealth and are willing to enroll in pragmatic trials evaluating MBSR.

**Objective:**

This study examined individual- and county-level social vulnerability characteristics associated with telehealth use experience among people living with CLBP who enrolled in OPTIMUM (Optimizing Pain Treatment in Medical Settings Using Mindfulness), a pragmatic randomized clinical trial of telehealth-delivered MBSR.

**Methods:**

We conducted a cross-sectional analysis of baseline measures from the OPTIMUM trial, focusing on participants from a safety-net health institution and a federally qualified health center. The primary outcome was telehealth use experience (having had a prior telehealth visit at or before enrollment). Guided by Andersen’s Behavioral Model of Health Services Use, we evaluated predisposing, enabling, and need factors. Prespecified measures included the following: demographics; employment and education; internet access and comfort using Zoom; neighborhood indices (Area Deprivation Index [ADI], Social Vulnerability Index [SVI]); rurality; back-pain duration; and validated patient-reported outcomes. Multivariable logistic regression was used to estimate adjusted odds ratios (AORs) and 95% CIs.

**Results:**

Of 244 enrolled participants (mean age 50.6, SD 13.5 y; n=162, 66.7% female; n=124, 50.8% non-Hispanic Black), 174 (71.3%) reported previous telehealth use. Predisposing factors associated with lower telehealth use included race or ethnicity (non-Hispanic Black: AOR 0.38, 95% CI 0.2‐0.8; Hispanic: AOR 0.21, 95% CI 0.06‐0.7) and part-time employment (AOR 0.33, 95% CI 0.1‐0.9) vs full-time employment, whereas educational attainment above or below a high school degree was associated with higher odds of telehealth use (AORs 2.7‐3.2). Enabling factors showed that lower comfort with Zoom was associated with lower telehealth use (AOR 0.5, 95% CI 0.2‐0.9), whereas home internet access was common and not independently significant in adjusted models. Neighborhood socioeconomic disadvantages, social vulnerability, and rurality did not demonstrate significant associations. Needs factors indicated that longer CLBP duration was positively associated with telehealth experience (>5 y: AOR 3.5, 95% CI 1.4‐8.8). Other patient-reported outcomes, comorbidity burden, and surgical history were not significantly associated.

**Conclusions:**

In a safety-net and federally qualified health center context, prior telehealth experience was common among patients with CLBP willing to enroll in a telehealth-delivered MBSR trial. Although certain predisposing factors (race or ethnicity, employment status) were associated with lower odds of telehealth experience, these factors did not prevent trial enrollment. Targeted outreach to improve digital comfort and inclusive engagement strategies may further reduce disparities and support broader implementation of telehealth for CLBP.

## Introduction

Chronic low back pain (CLBP) is a costly problem that results in US $100 to 200 billion per year in expenditures [1,2]. Many medical approaches used to treat CLBP are associated with adverse events and negative side effects [3,4]. Current treatment guidelines therefore recommend evidence-based, noninvasive approaches as first-line treatments for CLBP [5,6]. Mindfulness-based stress reduction (MBSR) is a recommended evidence-based treatment that focuses on increasing participants’ awareness and acceptance of moment-to-moment experiences, including physical discomfort and difficult emotions [7]. Prior research has found that medically underserved populations have an increased prevalence of chronic pain, less access to pain care, and poorer outcomes [8-11]. Telehealth-delivered MBSR offers a low-risk, effective, and viable alternative to conventional in-person delivery for patients with CLBP [12].

Telehealth refers to the use of electronic information and telecommunications (eg, videoconferencing) to provide clinical services [13]. Although prior to the COVID-19 pandemic, telehealth equipment was available, there was low usage due to a myriad of factors related to billing practices, patient and physician comfort, and familiarity with using the technology [14,15]. Use of telehealth leading up to the COVID-19 pandemic was more likely to be used by affluent, privately insured populations [16]. The COVID-19 pandemic created a unique and sudden need for virtual health visits for both urgent and nonurgent health visits, leading to an unprecedented expansion of telehealth access among underserved populations [17-19]. Expanded telehealth accessibility enhanced the potential to scale MBSR treatment to underserved populations.

In 2021, we launched OPTIMUM (Optimizing Pain Treatment In Medical Settings Using Mindfulness), a pragmatic randomized clinical trial comparing MBSR with usual care within primary care clinics for patients with CLBP at a safety-net hospital, federally qualified health centers, and a large academic health system. The COVID-19 pandemic created an urgent need for non–face-to-face care, prompting the conversion of MBSR from in-person to telehealth-delivered MBSR. While telehealth may improve access to MBSR, little data existed regarding how attractive this treatment would be to patients at safety net hospitals and federally qualified health centers. Safety-net hospitals and federally qualified health centers provide essential inpatient and outpatient services to low-income, uninsured people residing in rural areas and people with limited access to health care, that is, medically underserved populations [20].

Given the disparities and emerging telehealth opportunities, this study aimed to explore the impact of prior telehealth use on enrollment in the OPTIMUM trial. Therefore, the purpose of the study was to examine the individual- and county-level social vulnerability characteristics of people living with CLBP who were willing to enroll in OPTIMUM, a telehealth-delivered MBSR randomized pragmatic trial.

## Methods

### Theoretical Framework

Andersen’s Behavioral Model of Health Services Use has been widely used to analyze factors associated with health service use. The results suggest that health service usage is primarily motivated by individual illness, but the quality and quantity of health service usage vary significantly based on socioeconomic factors, such as income or health-insurance status [21-23]. The model posits 3 dimensions: predisposing factors (eg, age, education), enabling factors (eg, income, hospital density), and need factors (eg, health status) that influence health care usage [24-26]. Examining these dimensions may also be a suitable model when exploratory research is needed due to lack of previous studies on telehealth use among underserved patients with CLBP, as in this study.

### Study Design and Setting

This cross-sectional analysis was conducted on the baseline study measures data from the OPTIMUM trial, a multisite pragmatic randomized trial of telehealth-delivered MBSR for patients with CLBP. Data collection occurred between April 2021 and August 2023, with 12-month outcome data collection completed in November 2024. All variables were collected upon enrollment. OPTIMUM was embedded into primary care clinics at 3 health systems (Boston Medical Center, Massachusetts; University of Pittsburgh Medical Center, Pittsburgh, Pennsylvania; and Piedmont Health Services, in partnership with the University of North Carolina [UNC] Chapel Hill). The 3 health systems serve predominantly underserved patients: Boston Medical Center is a safety-net institution serving an urban, low-income, racially and ethnically diverse population. UNC Chapel Hill has 2 sites: an academic center–based family medicine clinic and a federally qualified health center network serving many rural patients and those underrepresented in research. The University of Pittsburgh serves an urban or suburban population. Because we were interested in the factors involved in telehealth use experience for underserved populations, we restricted our analysis to the baseline measurements of participants from the safety-net health setting (Boston Medical Center) and the federally qualified health centers (UNC at Chapel Hill, Piedmont Health Services).

### Recruitment

Research staff at each participating clinic approached potentially eligible patients during clinic visits, through telephone outreach, or via clinician referrals. Staff verified eligibility, provided study information, and obtained informed consent. Participants were screened to ensure they met inclusion criteria: age ≥18 years, diagnosis of CLBP, willing and able to provide telephone informed consent, able to speak and read English, absence of markers suggesting a serious condition, and a Pain, Enjoyment of Life and General Activity (PEG) scale score ≥3. Participants were excluded if they had an unexplained fever or weight loss, pregnancy, metastatic cancer, or cohabitation with another study participant.

### Participants of the OPTIMUM Study

Patients who met eligibility criteria were randomly assigned to medical group visits plus 90-minute, instructor-led MBSR treatment for 8 consecutive weeks via telehealth or to usual care alone. At the beginning of the MBSR session, for approximately 30 minutes, the primary care provider met briefly and privately with each participant through a telehealth visit (in a breakout room in the HIPAA [Health Insurance Portability and Accountability Act] compliant Zoom platform) to review medical aspects of treatment, focused specifically on the patient’s back pain. Participants were administered several measures at baseline, 8-week, 6-month, and the 12-month follow-up [27]. Further information about OPTIMUM is published elsewhere [27].

Because this analysis focused on understanding telehealth use experience among underserved populations, we restricted the analytic sample to participants enrolled from Boston Medical Center (safety-net setting) and UNC or Piedmont Health Services (federally qualified health centers).

### Outcome

Our outcome of interest in this paper was telehealth use experience as measured at enrollment, that is, telehealth use experience. Telehealth use experience includes the use of telehealth prior to or during the COVID-19 pandemic.

### Predictor Variables

The online baseline data included measures of demographics, back-related pain, treatments, the impact of pain on quality of life, pain beliefs, medical history, health habits, and traumatic experiences. Dimensions of Andersen’s Behavioral Model of Health Services Use and variables are as follows:

1. Predisposing factors: Age in 2021, sex, race or ethnicity, educational level, marital status, employment status, and health beliefs or psychological predispositions (Pain Catastrophizing Questionnaire [28], CAMS-R [Cognitive and Affective Mindfulness Scale-Revised] [29])
2. Enabling factors: Annual income, access to internet at home, comfort levels with Zoom or virtual meetings (confidence in their ability to use the platform), Area Deprivation Index (ADI), Social Vulnerability Index (SVI; both SVI and ADI are well-established indices that have been used to assess socioeconomic disadvantage and vulnerability [30,31]), and rural-urban commuting area
3. Needs factors: Number of years with low back pain; receiving disability or workers’ compensation due to low back pain or unemployed for 1 month or more due to low back pain [32]; Charlson Comorbidity Index [33]; number of low back surgeries; PROMIS-29 (Patient-Reported Outcomes Measurement Information System-29) domains, including sleep disturbance, fatigue, physical function, pain interference, ability to participate in social roles or activities, cognitive function, anxiety, and depression [34]; pain impact score; PEG [35]; TAPS (tobacco, alcohol, prescription medication, and other substance use) [36]

### Statistical Analyses

This study is reported in accordance with the STROBE (Strengthening the Reporting of Observational Studies in Epidemiology) guidelines (Checklist 1) [37] for cross-sectional studies. For descriptive analysis, we assessed the characteristics based on telehealth use experience (had previous telehealth visit or never had telehealth visit). Chi-square or Fisher exact tests were used for categorical variables, and Wilcoxon rank-sum tests were used for continuous variables.

Missing data were minimal (<5% for most variables). The low level of missingness did not warrant multiple imputation, which would have been unlikely to materially change the results. For continuous variables, histograms and Q-Q plots were used to assess normality. Nonnormally distributed variables were analyzed using nonparametric tests (Wilcoxon rank-sum). Categorical variables were analyzed using chi-square or Fisher exact tests depending on cell counts.

Bivariate analyses served as a screening step for multivariable modeling. A liberal threshold (*α*=.10) was used to avoid excluding potential predictors. Multivariable logistic regression retained variables with *P*<.05 in unadjusted models. Formal multiple-comparison correction was not applied because the primary goal was to build an adjusted model to identify factors associated with telehealth use experience, rather than to conduct multiple independent hypothesis tests.

The analytic sample was determined by available baseline data from the safety-net and federally qualified health center sites. These sites were selected based on the study objective of understanding telehealth use among underserved populations; thus, sample size was dictated by enrolled participants meeting eligibility criteria within these settings. We report both unadjusted odds ratios (ORs) and adjusted odds ratios (AORs) and 95% CIs as applicable. All analyses were conducted in SAS (version 9.4) with *P*<.05 considered statistically significant unless otherwise specified.

### Ethical Considerations

All study procedures were approved by the single Institutional Review Board at the University of Pittsburgh (STUDY20110378). All participants provided informed consent before the study and agreed to the use of their data for research purposes, including secondary analyses. Participation in the OPTIMUM study was voluntary, and participants could decline without consequences. To protect participants’ privacy and confidentiality, only anonymized data were used for this analysis, and results are reported in aggregate so that no individual participant can be identified. Participants were compensated US $30 for completing study measures administered at baseline and follow-up.

## Results

**Figure 1.**
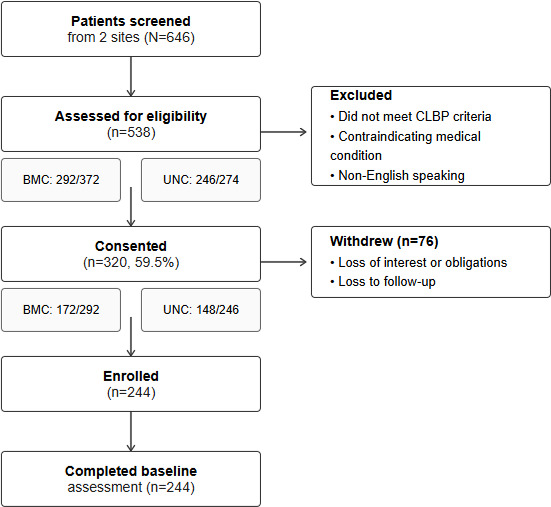
Participant flow diagram. CLBP: chronic low back pain; BMC: Boston Medical Center; UNC: University of North Carolina.

Out of 646 patients with CLBP from the safety-net health setting (Boston Medical Center) and the federally qualified health centers (UNC at Chapel Hill, Piedmont Health Services), 538 were assessed for eligibility, of whom 320 (59.5%) were eligible and consented. Of the 320 patients who consented, 244 completed baseline measures. Among those who initially enrolled but later withdrew, reasons included loss of interest, work or family obligations, or loss to follow-up. Patients who declined or were excluded were typically ineligible due to not meeting CLBP criteria, having contraindicated medical conditions, or being unable to complete consent in English (Figure 1). Analytic sample sizes were as follows: race or ethnicity (n=238), employment status (n=243), internet access (n=240), comfort with Zoom (n=241), and sex at birth (n=243). We performed a complete case analysis for multivariable modeling, yielding a final sample of 233.

Several factors were associated with having telehealth use experience. *Predisposing factors *such as race or ethnicity and employment status showed significant differences. Non-Hispanic White participants had the highest telehealth uptake at 84.1% (58/69), while Hispanic participants had the lowest at 56.5% (13/23; Table 1). In both the unadjusted and adjusted models (Tables 2 and 3), non-Hispanic Black participants had lower odds of telehealth use (OR 0.38, 95%  CI 0.2‐0.8). Hispanic participants also had lower odds in both models, with an unadjusted OR of 0.25 (95% CI 0.09‐0.7) and an AOR of 0.21 (95% CI 0.06‐0.7). Employment status was significantly associated with telehealth use experience (*P*=.03). Those who were employed part-time were less likely to have used telehealth (AOR 0.33, 95% CI 0.1‐0.9) in the adjusted model compared to those who worked full-time. Education demonstrated a positive trend, ranging from 62.3% (71/114) among those with a high school degree to 85.7% (12/14) among those with doctoral education, but the overall *P*=.07 was not significant. The pain catastrophizing questionnaire and CAMS-R were not significant (*P*=.40 and *P*=.87, respectively). Age, sex, and marital status were not significant predictors in the adjusted model (Table 3).

**Table 1.** Characteristics of OPTIMUM (Optimizing Pain Treatment In Medical Settings Using Mindfulness) study participants by telehealth group experience^a^.

Characteristics^b^^,^^c^	All (N=244)	Never had telehealth visit (n=70)	Had telehealth visit (n=174)	*P* value
Age (y), mean (SD)	50.6 (13.5)	50.8 (13.8)	50.5 (13.4)	.74^d^
Sex, n (%)				.09^e^
Male	81 (33.2)	29 (35.8)	52 (64.2)	
Female	162 (66.4)	41 (25.3)	121 (74.7)	
Race or ethnicity, n (%)				.04^f^^,^^g^
Non-Hispanic White	69 (28.3)	11 (15.9)	58 (84.1)	
Non-Hispanic Black	124 (50.8)	41 (33.1)	83 (66.9)	
Hispanic	23 (9.4)	10 (43.5)	13 (56.5)	
Non-Hispanic Other	13 (5.3)	5 (38.5)	8 (61.5)	
Unknown ethnicity, Black/African American	9 (3.7)	2 (22.2)	7 (77.8)	
Unknown or not reported	6 (2.5)	1 (16.7)	5 (83.3)	
Education, n (%)				.07^g^
Less than high school	6 (2.5)	1 (16.7)	5 (83.3)	
Some high school education	25 (10.2)	8 (32)	17 (68)	
High school diploma or equivalent	114 (46.7)	43 (37.7)	71 (62.3)	
Associate’s or technical degree	41 (16.8)	8 (19.5)	33 (80.5)	
College or baccalaureate degree	44 (18.0)	8 (18.2)	36 (81.8)	
Doctoral or postgraduate education	14 (5.7)	2 (14.3)	12 (85.7)	
Employment status, n (%)				.03^g^
Full-time	69 (28.3)	18 (26.1)	51 (73.9)	
Not employed	138 (56.6)	35 (25.4)	103 (74.6)	
Part-time	36 (14.8)	17 (47.2)	19 (52.8)	
Marital status, n (%)				.39^e^
Never married	98 (40.2)	35 (35.7)	63 (64.3)	
Married	44 (18.0)	12 (27.3)	32 (72.7)	
Divorced	46 (18.9)	11 (23.9)	35 (76.1)	
Separated	24 (9.8)	4 (16.7)	20 (83.3)	
Domestic partner	19 (7.8)	4 (21.1)	15 (78.9)	
Widowed	13 (5.3)	4 (30.8)	9 (69.2)	
Annual income (US $), n (%)				.40^g^
Less than 10,000	40 (16.4)	13 (32.5)	27 (67.5)	
10,000-24,999	56 (22.9)	14 (25)	42 (75)	
25,000-34,999	23 (9.4)	9 (39.1)	14 (60.9)	
35,000-49,999	29 (11.8)	6 (20.7)	23 (79.3)	
50,000-74,999	19 (7.8)	6 (31.6)	13 (68.4)	
75,000-99,999	6 (2.5)	3 (50)	3 (50)	
100,000-149,999	9 (3.7)	0 (0.0)	9 (100)	
150,000-199,999	2 (0.8)	0 (0.0)	2 (100)	
200,000 or more	1 (0.4)	0 (0.0)	1 (100)	
Prefer not to answer	56 (22.9)	17 (30.4)	39 (69.6)	
Years of low back pain, n (%)				<.001^f^
Less than 5 years	68 (27.9)	33 (48.5)	35 (51.5)	
5‐15 years	115 (47.1)	27 (23.5)	88 (76.5)	
More than 15 years	61 (25)	10 (16.4)	51 (83.6)	
Off work ≥1 month due to low back pain, n (%)				.68
Agree	112 (45.9)	30 (26.8)	82 (73.2)	
Disagree	78 (31.9)	22 (28.2)	56 (71.8)	
Does not apply	54 (22.1)	18 (33.3)	36 (66.7)	
Receives or has applied for disability or workers’ compensation due to low back pain, n (%)				.55
Agree	80 (32.8)	20 (25)	60 (75)	
Disagree	117 (47.9)	34 (29.1)	83 (70.9)	
Does not apply	47 (19.3)	16 (34.0)	31 (65.9)	
Home internet access with Zoom capability, n (%)				.04^g^
Yes	228 (93.4)	62 (27.2)	166 (72.8)	
No	12 (4.9)	7 (58.3)	5 (41.7)	
Comfort level with Zoom or virtual meetings, n (%)				.002^f^
Not at all	23 (9.4)	14 (60.9)	9 (39.1)	
A little bit	22 (9.0)	7 (31.8)	15 (68.2)	
Somewhat	24 (9.8)	8 (33.3)	16 (66.7)	
Quite a bit	67 (27.5)	11 (16.4)	56 (85.6)	
Very much	108 (44.3)	30 (27.8)	78 (72.2)	
Prior low back surgery, n (%)				.57^g^
Yes, 1 operation	26 (10.7)	7 (26.9)	19 (73.1)	
Yes, more than 1 operation	8 (3.3)	1 (12.5)	7 (87.5)	
No	210 (86.0)	62 (29.5)	148 (70.5)	
TAPS^h^ part 1: possible substance use, n (%)				.68^e^
Yes	166 (68.0)	49 (29.5)	117 (70.5)	
No	78 (31.9)	21 (26.9)	57 (73.1)	
PEG^i^ score, mean (SD)	6.7 (2.1)	7.1 (1.9)	6.5 (2.2)	.08^d^
Pain impact score, mean (SD)	32.2 (3.8)	32.8 (3.6)	32.0 (3.9)	.14^d^
PROMIS-29^j^ depression, mean (SD)	55.7 (9.4)	55.1 (9.7)	56.0 (9.3)	.57^d^
PROMIS-29 anxiety, mean (SD)	59.0 (9.1)	59.3 (10.3)	58.9 (8.6)	.80^d^
PROMIS-29 cognitive function, mean (SD)	47.1 (7.1)	46.7 (7.3)	47.1 (7.0)	.61^d^
PROMIS-29 ability to participate in social roles or activities, mean (SD)	44.2 (8.7)	44.8 (8.6)	43.9 (8.8)	.60^d^
PROMIS-29 pain interference, mean (SD)	63.7 (6.8)	63.4 (7.4)	63.8 (6.5)	.66^k^
PROMIS-29 physical function, mean (SD)	36.8 (5.6)	37.6 (5.4)	36.5 (5.7)	.27^d^
PROMIS-29 fatigue, mean (SD)	56.6 (9.7)	54.9 (9.5)	57.2 (9.7)	.10^k^
PROMIS-29 sleep disturbance, mean (SD)	58.0 (8.1)	58.5 (8.4)	57.8 (8.0)	.56^k^
Pain Catastrophizing Questionnaire, mean (SD)	13.8 (5.8)	14.3 (5.5)	13.7 (6.0)	.40^d^
CAMS-R^l^ mindfulness, mean (SD)	32.7 (6.8)	32.9 (7.5)	32.7 (6.6)	.87^d^
Charlson Comorbidity Index (weighted sum), mean (SD)	2.4 (3.3)	2.3 (2.7)	2.5 (3.5)	.64^d^
ADI^m^ national percentile (block group), quintile, n (%)				.27^e^
Quintile 1 (1‐20, least deprived)	90 (36.9)	31 (34.4)	59 (65.6)	
Quintile 2 (21-40)	69 (29.3)	21 (30.4)	48 (69.6)	
Quintile 3 (41-60)	22 (9.0)	3 (13.6)	19 (86.4)	
Quintile 4 (61-80)	25 (10.2)	8 (32)	17 (68)	
Quintile 5 (81‐100, most deprived)	30 (12.3)	6 (20)	24 (80)	
RUCA^n^ classification, n (%)				.88^e^
Metropolitan	222 (90.9)	64 (28.8)	158 (71.2)	
Nonmetropolitan or rural	22 (9.0)	6 (27.3)	16 (72.7)	

a Unless otherwise noted, percentages reflect row totals (ie, the percentage of participants with each characteristic who did vs did not have a telehealth visit).

b Percentages for age, PEG score, and other continuous measures are not applicable.

c Percentages are based on no missing data for each characteristic. The following variables had missing data (N=244 unless noted): sex (n=1 missing), employment status (n=1 missing), home internet access with Zoom capability (n=4 missing), ADI national percentile (n=8 missing).

d Wilcoxon rank-sum test.

e Chi-square test.

f Statistically significant at *P*<.05.

g Fisher exact test.

h TAPS: tobacco, alcohol, prescription medication, and other substance use.

i PEG: Pain, Enjoyment of Life, General Activity.

j PROMIS-29: Patient-Reported Outcomes Measurement Information System-29.

k Pooled 2-sample *t* test.

l CAMS-R: Cognitive and Affective Mindfulness Scale-Revised.

m ADI: Area Deprivation Index.

n RUCA: rural-urban commuting area.

**Table 2.** Univariate logistic regression of predictors of telehealth use (n=244).

Characteristic	Unadjusted	
	OR^a^ (95% CI)	*P* value
Sex at birth (n=243; reference: female)		
Male	0.6 (0.3‐1.1)	.09
Race or ethnicity (n=238; reference: Non-Hispanic White)		
Non-Hispanic Black	0.38 (0.2‐0.8)	.01^b^
Hispanic	0.25 (0.09‐0.7)	.008^b^
Other	0.41 (0.1‐1.2)	.11
Education (n=244; reference: high school degree)		
Less than high school	1.5 (0.6‐3.5)	.37
More than high school degree	2.7 (1.4‐5.1)	.002^b^
Employment status (n=243; reference: full-time)		
Not employed	1.03 (0.5‐2)	.91
Part-time	0.39 (0.2‐0.9)	.03^b^
Years of back pain (n=244; reference: less than 5 y)		
5 to 15 years	3.1 (1.6‐5.8)	<.001^b^
More than 15 years	4.8 (2.1‐11)	<.001^b^
Internet status: do you have internet at home and are you able to access programs like Zoom? (n=240; reference: no)		
Yes	3.7 (1.1‐12.2)	.02^b^
Comfort levels with Zoom or virtual meetings (n=241; reference: high comfort level)		
Low comfort level	0.4 (0.2‐0.7)	.004^b^
PEG^c^ (n=244)	0.8 (0.7‐1.01)	.08
PROMIS^d^-29 fatigue (n=244)	1.02 (0.9‐1.05)	.10

a OR: odds ratio.

b Statistically significant at *P*<.05.

c PEG: Pain, Enjoyment of Life, and General Activity.

d PROMIS-29: Patient-Reported Outcomes Measurement Information System-29.

**Table 3.** Adjusted logistic regression of predictors of telehealth use (N=233).

Characteristic	Adjusted	
	OR^a^ (95% CI)	*P* value
Race or ethnicity (reference: Non-Hispanic White)		
Non-Hispanic Black	0.38 (0.2‐0.8)	.02^b^
Hispanic	0.21 (0.06‐0.7)	.01^b^
Other	0.32 (0.1‐1.1)	.07
Education (reference: high school degree)		
Less than high school	3.2 (1.1‐8.9)	.03^b^
More than high school degree	2.7 (1.3‐5.1)	.01^b^
Employment status (reference: full-time)		
Not employed	1.11 (0.5‐2.4)	.91
Part-time	0.33 (0.1‐0.9)	.03^b^
Years of back pain (reference: less than 5 y)		
5 to 15 years	3.1 (1.6‐6.5)	<.001^b^
More than 15 years	3.5 (1.4‐8.8)	<.001^b^
Internet status: do you have internet at home and are you able to access programs like Zoom? (reference: no)		
Yes	2.2 (0.6‐8.7)	.25
Comfort levels with Zoom or virtual meeting (reference: high comfort level)		
Low comfort level	0.5 (0.2‐0.9)	.04^b^

a OR: odds ratio.

b Statistically significant at *P*<.05.

Among *enabling factors*, access and comfort with technology were strongly associated with telehealth use. Participants who had internet at home and had the ability to use Zoom had a telehealth usage rate of 72.8% compared to 41.7% among those without access (*P*=.04). Comfort with Zoom ranged from “not at all” 39.1% (9/23), to “quite a bit” 83.6% (56/67), to “very much” comfortable 72.2% (78/108); lower comfort levels were associated with lower odds of telehealth use experience (AOR 0.5, 95% CI 0.2‐0.9). Most of the sample, 169 of 236 (71.6%) participants, were in the low ADI (≤50) national percentile block group, indicating neighborhoods with lower levels of socioeconomic disadvantage. The mean SVI score was 0.7 (SD 0.3), indicating a moderate-to-high level of vulnerability. See Multimedia Appendix 1 for ADI and SVI quartiles, quintiles, mean scores, and specific cutoff points (ie, >50th percentile vs <50th percentile). Overall, annual income, ADI, SVI, and rurality (metro vs nonmetro or rural) were not significant factors associated with telehealth use experience in both the unadjusted and the adjusted models.

Among *needs factors,* years with back pain strongly predicted telehealth use experience: 51.5% (35/68) among those with less than 5 years, 76.5% (88/115) among those with 5 to 15 years, and 83.6% among those with more than 15 years of pain (*P*<.001). In the adjusted model, more than 5 years of pain was associated with higher odds of telehealth use experience (AOR 3.5, 95% CI 1.4‐8.8). Having received or applying for disability or workers’ compensation benefits due to low back pain was not a significant factor in telehealth use. Other need indicators such as PEG, pain impact score, PROMIS-29 domains (sleep disturbance, fatigue, physical function, pain interference, ability to participate in social roles or activities, cognitive function, anxiety, and depression), comorbidity index, and lower back operations were not significantly associated with telehealth use experience.

## Discussion

### Principal Findings

This cross-sectional analysis examined the individual- and county-level social vulnerability characteristics of those who had and those who never had a telehealth visit at enrollment among participants from a safety-net hospital and a network of federally qualified health centers in the OPTIMUM trial. We found that 174 of 244 (71.3%) participants had prior experience with telehealth. While a large portion of our sample had previously used telehealth, there were many who had not, thus indicating that while telehealth may be well received by many patients, it remains more accessible to certain groups of patients than others [17,38].

Similar to previous research, we found that females reported a higher experience with telehealth usage compared to males [19]. Our analysis indicated that compared to non-Hispanic White participants, non-Hispanic Black and Hispanic participants had lower odds of telehealth use experience. Given the range of health disparities across the United States, it is reasonable to assume that not all people benefit from using telehealth. In the pre–COVID-19 period, telehealth visits were significantly lower among Hispanic patients than among non-Hispanic White patients and non-Hispanic Black patients [39]. Although telehealth use increased among these groups during the COVID-19 period, disparities persist with reliable internet connectivity, digital literacy, and user comfort with technology among these populations [40,41].

In the adjusted models, we did not find a statistically significant association in our data between internet access and telehealth use experience, which may be explained by factors unique to our sample, such as high number of participants with internet access (228/244, 93.4%) and a large portion of our sample (213/244, 87.3%) having a high school degree or higher, which may have impacted significance. Previous research has found that while internet access is a foundational requirement for telehealth, it is not the only or even the most significant factor determining telehealth use when compared to, or in combination with, education, employment status, and digital literacy [42,43].

Unsurprisingly, lower comfort with Zoom and other virtual meeting platforms was associated with markedly lower odds of telehealth use experience. Prior research has found that the strongest predictor for telehealth use among participants was their comfort with technology rather than access alone [44]. Additionally, we found that in comparison to a high school degree, having less than a high school degree or more than a high school degree was associated with greater odds of previous telehealth use after adjustment. Our findings add to the mixed results of previous studies, which have found either no differences associated with telehealth use or that lower education and income were associated with lower telehealth use [45,46].

Our findings indicated that participants who had 5 or more years of CLBP had higher telehealth use experience, which may have influenced their desire to participate in the telehealth trial. Previous research has shown that patients with chronic pain are more likely to use telehealth because it offers easy access to specialists and reduces the amount of time taken off work to travel to an appointment [47-49].

We found no statistically significant association between ADI, SVI ranking, and the odds of previous telehealth use. Characteristics of our sample may explain possible reasons why ADI and SVI ranking was not significantly associated with previous telehealth. Among 244 participants, a large portion of our sample (n=213, 87.3%) had a high school degree or higher, lived in the metro area (n=222, 90.9%), and had access to the internet at home (n=228, 93.4%). Other research on the association between ADI, SVI, and telehealth usage shows mixed results. While some studies link higher disadvantage (higher ADI or SVI) to lower telehealth usage, others report no significant association between these indices and telehealth, suggesting factors such as age or technology access may play a larger role [50-52].

### Strengths and Limitations

Our study has several strengths and limitations. The strengths of our study include a large sample size of an understudied population with CLBP. Additional strengths include the use of reliable and valid measurements. A limitation of this study is that all participants voluntarily enrolled in a telehealth trial and were required to have internet access in some form, which may indicate a higher comfort level with technology and limit the generalizability of the findings. Additionally, participants in our sample were more likely to receive a telehealth visit because they lived in communities with higher telehealth use (eg, urban communities served by large health systems).

Another limitation was the comfort with Zoom or virtual meeting platforms measure, which captured participants’ confidence in using these platforms. The measure did not discern whether participants had adequate digital literacy skills, just if they felt confident using those platforms. Other limitations include the following: (1) the cross-sectional nature of the study design, which precludes any ability to draw causal inferences; (2) self-reported variables, introducing the likelihood of recall bias; and (3) the recruitment of most participants from metro areas. Prior research has found that while both metro and rural underserved populations own and use digital health technology, rural residents are less likely to communicate with their health providers using that technology [53].

### Implications and Recommendations

The results of our study have important implications. First, given the high economic burden of CLBP and the growing emphasis on evidence-based treatments such as MBSR, telehealth offers a promising alternative for medically underserved populations. Factors that served as enablers included home internet access and greater comfort with Zoom. Targeted outreach and culturally tailored engagement strategies are needed to address disparities related to race or ethnicity and employment status. Policymakers and health care stakeholders must take proactive measures to mitigate these disparities, enhance access, and ensure that telehealth fulfills its potential to improve outcomes for all patients with CLBP.

### Conclusions

In a safety-net and federally qualified health center context, prior telehealth experience was common among patients with CLBP willing to enroll in a telehealth-delivered MBSR trial. Although certain predisposing factors (race or ethnicity, employment status) were associated with lower odds of telehealth experience, these factors did not prevent trial enrollment. Thus, targeted outreach to improve digital comfort and inclusive engagement strategies may further reduce disparities and support broader implementation of telehealth for CLBP.

## Supplementary material

10.2196/82093 Multimedia Appendix 1 Different ways to interpret Area Deprivation Index and Social Vulnerability Index.

10.2196/82093 Checklist 1 STROBE checklist.

## Abbreviations

ADI: Area Deprivation Index
AOR: adjusted odds ratio
CAMS-R: Cognitive and Affective Mindfulness Scale-Revised
CLBP: chronic low back pain
HIPAA: Health Insurance Portability and Accountability Act
MBSR: mindfulness-based stress reduction
OPTIMUM: Optimizing Pain Treatment in Medical Settings Using Mindfulness
OR: odds ratio
PEG: Pain, Enjoyment of Life, and General Activity
PROMIS-29: Patient-Reported Outcomes Measurement Information System-29
STROBE: Strengthening the Reporting of Observational Studies in Epidemiology
SVI: Social Vulnerability Index
TAPS: tobacco, alcohol, prescription medication, and other substance use
UNC: University of North Carolina

## References

[1] Goode AP, Cleveland RJ, Schwartz TA (2019). Relationship of joint hypermobility with low back pain and lumbar spine osteoarthritis. BMC Musculoskelet Disord.

[2] (2025). 57 back pain statistics-understanding the prevalence and impact of back pain: a statistical overview. April ABA.

[3] Morlion B (2013). Chronic low back pain: pharmacological, interventional and surgical strategies. Nat Rev Neurol.

[4] Deyo RA, Mirza SK, Turner JA, Martin BI (2009). Overtreating chronic back pain: time to back off?. J Am Board Fam Med.

[5] Day MA, Ciol MA, Mendoza ME (2024). The effects of telehealth-delivered mindfulness meditation, cognitive therapy, and behavioral activation for chronic low back pain: a randomized clinical trial. BMC Med.

[6] Qaseem A, Wilt TJ, McLean RM (2017). Noninvasive treatments for acute, subacute, and chronic low back pain: a clinical practice guideline from the American College of Physicians. Ann Intern Med.

[7] Kabat-Zinn J (2009). Full Catastrophe Living Using the Wisdom of Your Body and Mind to Face Stress, Pain, and Illness.

[8] Baker MB, Liu EC, Bully MA (2024). Overcoming barriers: a comprehensive review of chronic pain management and accessibility challenges in rural America. Healthcare (Basel).

[9] Nguyen LH, Dawson JE, Brooks M, Khan JS, Telusca N (2023). Disparities in pain management. Anesthesiol Clin.

[10] Cyr ME, Etchin AG, Guthrie BJ, Benneyan JC (2019). Access to specialty healthcare in urban versus rural US populations: a systematic literature review. BMC Health Serv Res.

[11] Burke CA, Fillipo R, Epplein M, Brookhart MA, Bosworth HB, Goode AP (2026). Racial and ethnic disparities in the incidence and prevalence of low back pain in the United States: a systematic review. Arthritis Care Res (Hoboken).

[12] Chou R, Qaseem A, Snow V (2007). Diagnosis and treatment of low back pain: a joint clinical practice guideline from the American College of Physicians and the American Pain Society. Ann Intern Med.

[13] (2019). What is telehealth? How is telehealth different from telemedicine?. HealthIT.gov.

[14] Grigsby B, Brega AG, Bennett RE (2007). The slow pace of interactive video telemedicine adoption: the perspective of telemedicine program administrators on physician participation. Telemed J E Health.

[15] Al Mohaya MA, Almaziad MM, Al-Hamad KA, Mustafa M (2021). Telemedicine among oral medicine practitioners during COVID-19 pandemic and its future impact on the specialty. Risk Manag Healthc Policy.

[16] Barnhill JL, Castro G, Lathren C (2025). The hidden complexity of virtual mindfulness-based group medical visits: comfort, challenge, and the influence of social determinants of health. Glob Adv Integr Med Health.

[17] Franciosi EB, Tan AJ, Kassamali B (2021). The impact of telehealth implementation on underserved populations and no-show rates by medical specialty during the COVID-19 pandemic. Telemed J E Health.

[18] Williams C, Shang D (2023). Telehealth usage among low-income racial and ethnic minority populations during the COVID-19 pandemic: retrospective observational study. J Med Internet Res.

[19] Xu P, Hudnall M, Zhao S, Raja U, Parton J, Lewis D (2022). Pandemic-triggered adoption of telehealth in underserved communities: descriptive study of pre- and postshutdown trends. J Med Internet Res.

[20] Popescu I, Fingar KR, Cutler E, Guo J, Jiang HJ (2019). Comparison of 3 safety-net hospital definitions and association with hospital characteristics. JAMA Netw Open.

[21] Kim HK, Lee M (2016). Factors associated with health services utilization between the years 2010 and 2012 in Korea: using Andersen’s behavioral model. Osong Public Health Res Perspect.

[22] Andersen R, Lewis SZ, Giachello AL, Aday LA, Chiu G (1981). Access to medical care among the Hispanic population of the southwestern United States. J Health Soc Behav.

[23] Gelberg L, Andersen RM, Leake BD (2000). The behavioral model for vulnerable populations: application to medical care use and outcomes for homeless people. Health Serv Res.

[24] Andersen RM (2008). National health surveys and the behavioral model of health services use. Med Care.

[25] Babitsch B, Gohl D, von Lengerke T (2012). Re-revisiting Andersen’s Behavioral Model of Health Services Use: a systematic review of studies from 1998-2011. Psychosoc Med.

[26] Andersen RM, Davidson PL, Baumeister SE (2007). Changing the US Health Care System: Key Issues in Health Services Policy and Management.

[27] Greco CM, Gaylord SA, Faurot K (2021). The design and methods of the OPTIMUM study: a multisite pragmatic randomized clinical trial of a telehealth group mindfulness program for persons with chronic low back pain. Contemp Clin Trials.

[28] McWilliams LA, Kowal J, Wilson KG (2015). Development and evaluation of short forms of the Pain Catastrophizing Scale and the Pain Self-efficacy Questionnaire. Eur J Pain.

[29] Feldman G, Hayes A, Kumar S, Greeson J, Laurenceau JP (2007). Mindfulness and emotion regulation: the development and initial validation of the Cognitive and Affective Mindfulness Scale-Revised (CAMS-R). J Psychopathol Behav Assess.

[30] Spielman SE, Tuccillo J, Folch DC (2020). Evaluating social vulnerability indicators: criteria and their application to the Social Vulnerability Index. Nat Hazards.

[31] Kind AJH, Jencks S, Brock J (2014). Neighborhood socioeconomic disadvantage and 30-day rehospitalization: a retrospective cohort study. Ann Intern Med.

[32] Beneciuk JM, Bishop MD, Fritz JM (2013). The STarT back screening tool and individual psychological measures: evaluation of prognostic capabilities for low back pain clinical outcomes in outpatient physical therapy settings. Phys Ther.

[33] Charlson M, Szatrowski TP, Peterson J, Gold J (1994). Validation of a combined comorbidity index. J Clin Epidemiol.

[34] Dewitt B, Feeny D, Fischhoff B (2018). Estimation of a preference-based summary score for the Patient-Reported Outcomes Measurement Information System: the PROMIS®-Preference (PROPr) scoring system. Med Decis Making.

[35] Krebs EE, Lorenz KA, Bair MJ (2009). Development and initial validation of the PEG, a three-item scale assessing pain intensity and interference. J Gen Intern Med.

[36] McNeely J, Wu LT, Subramaniam G (2016). Performance of the Tobacco, Alcohol, Prescription Medication, and Other Substance Use (TAPS) tool for substance use screening in primary care patients. Ann Intern Med.

[37] von Elm E, Altman DG, Egger M (2007). The Strengthening the Reporting of Observational Studies in Epidemiology (STROBE) statement: guidelines for reporting observational studies. PLoS Med.

[38] Shaver J (2022). The state of telehealth before and after the COVID-19 pandemic. Prim Care.

[39] White-Williams C, Liu X, Shang D, Santiago J (2023). Use of telehealth among racial and ethnic minority groups in the United States before and during the COVID-19 pandemic. Public Health Rep.

[40] Campos-Castillo C, Anthony D (2021). Racial and ethnic differences in self-reported telehealth use during the COVID-19 pandemic: a secondary analysis of a US survey of internet users from late March. J Am Med Inform Assoc.

[41] Iasiello JA, Rajan A, Zervos E, Parikh AA, Snyder RA (2023). Racial differences in patient-reported access to telehealth: an important and unmeasured social determinant of health. JCO Oncol Pract.

[42] Labadorf J, Nichols M, Williams T, Cunanan C, D’Anza B (2025). A smartphone is not enough: telehealth attendance and the digital divide. Health Care Sci.

[43] Kemp M, Rising KL, Laynor G (2025). Barriers to telehealth uptake and use: a scoping review. JAMIA Open.

[44] Yamazaki KG, Hewitt L, Torres L, Colon-Vazquez K, Rogers P, Wang G (2026). Technology access, digital literacy, and enrollment support preferences in a federally qualified health center: cross-sectional study. JMIR Form Res.

[45] Thomson MD, Mariani AC, Williams AR, Sutton AL, Sheppard VB (2021). Factors associated with use of and satisfaction with telehealth by adults in rural Virginia during the COVID-19 pandemic. JAMA Netw Open.

[46] Kini S, Duluk D, Weinstein J (2023). Modeling the impact of digital readiness in recruiting and sustaining underrepresented groups: data from the All of Us research program. Front Digit Health.

[47] Tauben DJ, Langford DJ, Sturgeon JA (2020). Optimizing telehealth pain care after COVID-19. Pain.

[48] Pritzlaff SG, Singh N, Sanghvi C, Schatman ME (2025). Telehealth is crucial for pain medicine: patients and doctors are at the brink, and Medicare must act now. J Pain Res.

[49] McLaughlin KH, Fritz JM, Minick KI (2024). Examining the relationship between individual patient factors and substantial clinical benefit from telerehabilitation among patients with chronic low back pain. Phys Ther.

[50] Bose S, Dun C, Zhang GQ, Walsh C, Makary MA, Hicks CW (2022). Medicare beneficiaries in disadvantaged neighborhoods increased telemedicine use during the COVID-19 pandemic. Health Aff (Millwood).

[51] Boudreau E, Sutherland A, Bozzi D, Canterberry M, Sylwestrzak G (2024). Primary care telehealth utilization by access-challenged populations in Medicare Advantage. Health Aff Sch.

[52] Ostovari M, Zhang Z, Patel V, Jurkovitz C (2023). Telemedicine and health disparities: association between the area deprivation index and primary care telemedicine utilization during the COVID-19 pandemic. J Clin Transl Sci.

[53] Okobi E, Adigun AO, Ozobokeme OE (2023). Examining disparities in ownership and use of digital health technology between rural and urban adults in the US: an analysis of the 2019 Health Information National Trends Survey. Cureus.

