# Supplementary material for: The Impact of Telehealth Use Experience on Recruitment of Underserved Populations Into a Telehealth-Delivered Mindfulness-Based Trial for Patients With Chronic Low Back Pain: Cross-Sectional Survey
Source: JMIR Mhealth Uhealth. 2026 Jul 29;14:e82093. doi: 10.2196/82093 (PMC13418554; doi:10.2196/82093)
Supplement: Multimedia Appendix 1 [file mhealth-v14-e82093-s001.docx]

| **Supplement Table 1. Different ways to interpret Area Deprivation Index &** **Social Vulnerability Index** | | | | |
| --- | --- | --- | --- | --- |
| **Characteristic** | **All**  **(n=244)** | **Never had a telehealth visit (n=70)** | **Previously had a telehealth visit**  **(n=174)** | **P value** |
| **National percentile of block group ADI score – Quintiles**, No. (%) |  |  |  | 0.27^a^ |
| Quintile 1 (1-20) | 90 (38.1) | 31 (34.4) | 59 (65.6) |  |
| Quintile 2 (21-40) | 69 (29.2) | 21 (30.4) | 48 (69.6) |  |
| Quintile 3 (41-60) | 22 (9.3) | 3 (13.6) | 19 (86.4) |  |
| Quintile 4 (61-80) | 25 (10.6) | 8 (32) | 17 (68) |  |
| Quintile 5 (81-100) | 30 (12.7) | 6 (20) | 24 (80) |  |
| **National percentile of block group ADI score - Quartiles**, No. (%) |  |  |  | 0.73^a^ |
| Quartile 1 (1-24) | 111 (47) | 35 (31.5) | 76 (68.5) |  |
| Quartile 2 (25-49) | 58 (24.6) | 18 (31) | 40 (69) |  |
| Quartile 3 (50-74) | 30 (12.7) | 7 (23.3) | 23 (76.7) |  |
| Quartile 4 (75-100) | 37 (15.7) | 9 (24.3) | 28 (75.7) |  |
| **National percentile of block group ADI score**, No. (%) |  |  |  | 0.25^a^ |
| Low ADI (≤ 50) | 169 (71.6) | 53 (31.4) | 116 (68.6) |  |
| High ADI (>50) | 67 (28.4) | 16 (23.9) | 51 (76.1) |  |
| **National percentile of block group ADI score** | 236 | 69 | 167 | 0.13^b^ |
| Mean (SD) | 36.6 (27.1) | 32.7 (25.5) | 38.2 (27.6) |  |
| **Social vulnerability index score – Quartile,** No. (%) |  |  |  | 0.99^a^ |
| Quartile 1 (0-0.25) | 30 (12.6) | 8 (26.7) | 22 (73.3) |  |
| Quartile 2 (>0.25-0.50) | 34 (14.3) | 10 (29.4) | 24 (70.6) |  |
| Quartile 3 (>0.50-0.75) | 56 (23.5) | 16 (28.6) | 40 (71.4) |  |
| Quartile 4 (>0.75-1.00) | 118 (49.6) | 35 (29.7) | 83 (70.3) |  |
| **Social vulnerability index score** | 238 | 69 | 169 | 0.64^b^ |
| Mean (SD) | 0.7 (0.3) | 0.67 (0.3) | 0.66 (0.3) |  |

^a^Chi-square test. ^b^Wilcoxon rank-sum test.
